# Recurrent Aggressive Osteoblastoma of the Cervical Spine Successfully Treated with Radiotherapy

**DOI:** 10.1055/s-0041-1739300

**Published:** 2021-11-16

**Authors:** Gabriel Marques dos Anjos, Marcos Antonio Freiberger Bassan, Gustavo Gössling, Fernando de Souza Pereira, Rui D'Avila, Patrícia Izetti

**Affiliations:** 1 Instituto de Oncologia Kaplan, Porto Alegre, RS, Brasil; 2 Hospital Ernesto Dornelles, Porto Alegre, RS, Brasil; 3 Vitta Centro Avançado de Radioterapia, Porto Alegre, RS, Brasil

**Keywords:** cervical vertebrae, osteoblastoma, radiotherapy, spinal neoplasms

## Abstract

Osteoblastoma is a benign disease usually treated with complete resection with curative intent, and it accounts for 14% of the benign bone tumors. A more uncommon presentation refers to a rapid growing mass known as aggressive osteoblastoma. We report the case of a young male with cervical swelling due to a progressive mass on the right side of neck with 3 months of evolution. Incisional biopsy demonstrated grade 3 osteoblastoma. A radical surgical resection was attempted, but complete resection was not feasible because of cervical invasion of C3 without spinal involvement. Despite the lack of robust data, intensity modulated radiotherapy was performed in the ipsilateral cervical region at a dose of 50 Gy in 25 fractions until November 2013. The patient is being followed up regularly without evidence of recurrence since. The so far successful treatment highlights the importance of a multidisciplinary approach for treating patients with aggressive osteoblastoma.

## Introduction

Osteoblastoma is a benign disease usually treated with complete resection with curative intent, and it accounts for 14% of the benign bone tumors. 1 Aggressive osteoblastoma (AO) is a rare form of presentation, with no malignant features on microscopic evaluation despite higher recurrence rates and capacity of malignant transformation when compared with classic osteoblastoma. 2 The standard treatment consists of extensive resection, mainly because of its destructive clinical evolution, and it is potentially associated with higher surgical complications. In the present report, we describe the case of an unresectable aggressive osteoblastoma of the cervical spine treated successfully with radiotherapy.

## Case Report

**Fig. 1:**
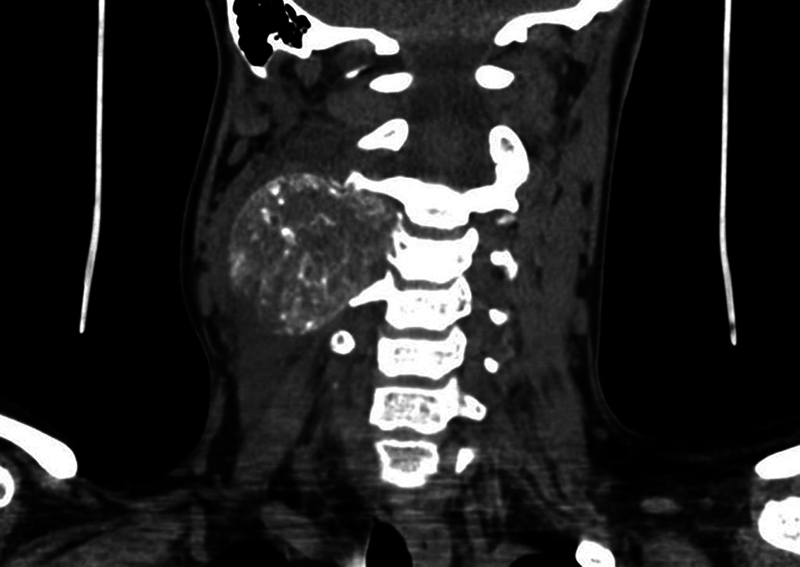
Computed tomography with C3 involvement at diagnosis.

**Fig. 2:**
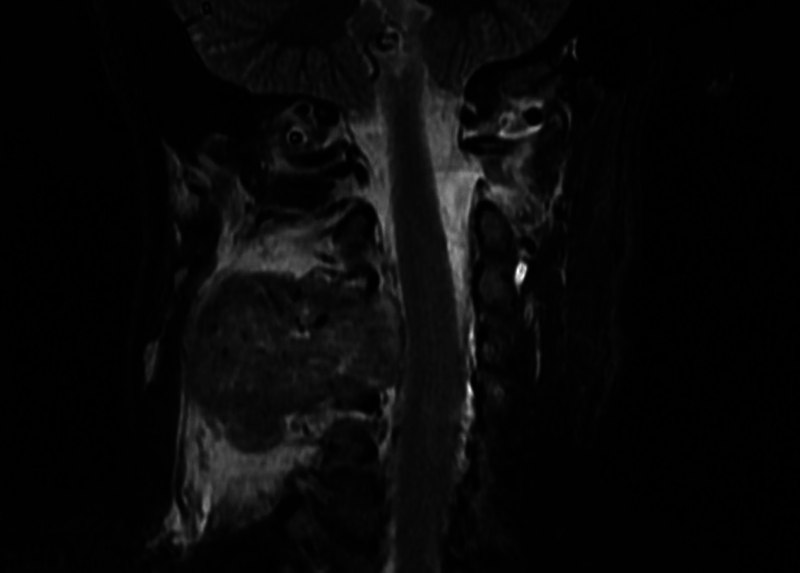
Magnetic resonance imaging with C3 involvement without spinal compromise.

We report the case of a young male with cervical swelling due to a progressive mass on the right side of the neck with 3 months of evolution. Incisional biopsy demonstrated grade 3 osteoblastoma. A radical surgical resection was attempted, but complete resection was not feasible because of cervical invasion of C3 without spinal involvement ( Figs. 1 and 2 ).

The surgical procedure was deemed incomplete again, due to more extensive involvement of the vertebral bone, now also compromising C3. After multidisciplinary discussion, the potential risks of chemotherapy, radiotherapy, and another surgical procedure aiming for local control were weighted. Despite the lack of robust data, intensity modulated radiotherapy was performed in the ipsilateral cervical region at a dose of 50 Gy in 25 fractions until November 2013. The patient is being followed up regularly without evidence of recurrence. He has a sequela involving limitation of strength in the shoulder, mainly due to loss of musculature associated with the surgical procedure. Despite this, the patient has a normal life and can practice sports often.

## Discussion

Osteoblastoma corresponds to 1% of all primary bone neoplasms. It classically affects more males than females, at a 2:1 ratio, and it is diagnosed in the first 3 decades of life. 3 The more commonly affected bones are the spinal column, 9% on the cervical segment, and long bones. 4 Recurrence rates for AO are as high as 50%. 2 The clinical presentation is diverse when related to the affected area, including many asymptomatic cases on diagnosis. 4 In our case, the patients' complaints were swelling with rapid growing on the cervical area, facilitating the diagnosis.

One of the main challenges is to differentiate AO from osteosarcoma, with a quarter of cases having radiologic features of malignancy, similar to low grade osteosarcoma. There is such an overlap of histologic and radiologic features that clinical course plays a critical role. 4

Osteoblastomas are usually histologically indistinguishable from osteoid osteomas, with the latter rarely being bigger than 1.5 cm. Incidence rates on the spine are 40%, usually compromising the pedicles and the lamina. On the other hand, AO is associated more often with the paravertebral and epidural extension.

Aggressive osteoblastoma may also be similar to aneurysmal bone cysts, osteosarcomas and bone metastases, presenting with an expansible pattern with matrix calcification and cortical bone destruction. 2

Osteoblastomas are, usually, radiolucent lesions, poorly defined with cortical destruction or well-defined borders and different degrees of mineralization. 5 Histopathologically, AO presents with nucleoli prominence, larger and irregular trabeculae, eosinophilic cytoplasm, and tendency to have more osteoclast-like cells. Mild cellular pleomorphism and mitoses are more often seen. 2

Generally, treatment of this aggressive form of disease is based on wide and complete resection of the primary tumor. Additional complication comes when the primary site is close to noble structures, like in this case, with involvement of the cervical spine. Standard of care is still considered a radical and complete resection, 6 but there are case reports of the potential efficacy of radiotherapy, with 25 years being the longest time in remission published. A successful treatment with curettage and radiotherapy is described in a case in which the resection was considered impossible. Janin et al. 7 states that radiotherapy should be considered only for patients whose lesions are unresectable. There are some case reports associating radiotherapy for AO with the development of sarcoma, leading to death in the worst scenario. 8 9 This worrisome complication is aggravated when radiotherapy is used, especially in benign lesions.

The present report demonstrates a clinical demand for randomized trials on rare diseases. There are several designs and efforts that can be done to overcome the natural problem of testing new treatments in the context of rare diseases. 2 This report adds to the clinical data that radiotherapy is a potential treatment for aggressive osteoblastoma. In addition, there are reports of cases with recurrence of osteoblastoma after radiotherapy similar to this case. 10 The so far successful treatment highlights the importance of a multidisciplinary approach for treating these patients, especially in cases of rare diseases.
