# Supplementary material for: Recurrent Aggressive Osteoblastoma of the Cervical Spine Successfully Treated with Radiotherapy
Source: Rev Bras Ortop (Sao Paulo). 2021 Nov 16;59(Suppl 2):e138–40. doi: 10.1055/s-0041-1739300 (PMC11679614; doi:10.1055/s-0041-1739300)
Supplement: Related texts

# Research article: Osteoblastoma agressivo recorrente da coluna cervical tratado com sucesso com radioterapia

**Keywords:** neoplasias da coluna vertebral, osteoblastoma, radioterapia, vértebras cervicais

## Abstract

O osteoblastoma é uma doença benigna geralmente tratada com ressecção completa com intenção curativa e é responsável por 14% dos tumores ósseos benignos. Uma apresentação mais incomum refere-se a uma rápida apresentação em massa conhecida como osteoblastoma agressivo. Relatamos o caso de um jovem com inchaço cervical devido a uma massa progressiva no lado direito do pescoço com 3 meses de evolução. A biópsia incisional demonstrou osteoblastoma grau 3. Tentou-se uma ressecção cirúrgica radical, mas a ressecção completa não foi viável por causa da invasão cervical de C3 sem envolvimento espinal. Apesar da falta de dados robustos, a radioterapia modulada de intensidade foi realizada na região cervical ipsilateral em uma dose de 50 Gy em 25 frações até novembro de 2013. O paciente está sendo seguido regularmente sem evidências de recorrência desde então. O tratamento até agora bem-sucedido destaca a importância de uma abordagem multidisciplinar para o tratamento de pacientes com osteoblastoma agressivo.

## Introdução

O osteoblastoma é uma doença benigna geralmente tratada com ressecção completa com intenção curativa e é responsável por 14% dos tumores ósseos benignos. 1 O osteoblastoma agressivo (OA) é uma forma rara de apresentação, sem características malignas na avaliação microscópica, apesar das maiores taxas de recorrência e capacidade de transformação maligna quando comparada com o osteoblastoma clássico. 2 O tratamento padrão consiste em ressecção extensiva, principalmente por causa de sua evolução clínica destrutiva e está potencialmente associado a complicações cirúrgicas mais elevadas. Neste relatório, descrevemos um caso de osteoblastoma agressivo não ressecável da coluna cervical tratado com sucesso com radioterapia.

## Relatório de Caso

Relatamos o caso de um jovem com inchaço cervical devido a uma massa progressiva no lado direito do pescoço com 3 meses de evolução. A biópsia incisional demonstrou osteoblastoma grau 3. Tentou-se uma ressecção cirúrgica radical, mas a ressecção completa não foi viável por causa da invasão cervical de C3 sem envolvimento espinal ( Figs. 1 e 2 ).

**Fig. 1:** Tomografia computadorizada com envolvimento de C3 no diagnóstico.

**Fig. 2:** Ressonância magnética com envolvimento de C3 sem comprometimento espinal.

O procedimento cirúrgico foi considerado incompleto novamente, devido ao envolvimento mais extenso do osso vertebral, comprometendo também C3. Após discussão multidisciplinar, foram ponderados os riscos potenciais da quimioterapia, da radioterapia e de outro procedimento cirúrgico visando o controle local. Apesar da falta de dados robustos, a radioterapia modulada de intensidade foi realizada na região cervical ipsilateral em uma dose de 50 Gy em 25 frações até novembro de 2013. O paciente está sendo seguido regularmente sem evidências de recorrência. Ele tem uma sequela envolvendo limitação da força do ombro, principalmente devido à perda de musculatura associada ao procedimento cirúrgico. Apesar disso, o paciente tem uma vida normal e pode praticar esportes com frequência.

## Discussão

O osteoblastoma corresponde a 1% de todas as neoplasias ósseas primárias. Ela afeta mais o sexo masculino do que o feminino, em uma proporção de 2:1, e é diagnosticada nas três primeiras décadas de vida. 3 Os ossos mais comumente afetados são a coluna vertebral, 9% no segmento cervical, e ossos longos. 4 As taxas de recorrência do OA são de até 50%. 2 A apresentação clínica é diversificada quando relacionada à área afetada, incluindo muitos casos assintomáticos no diagnóstico. 4 No nosso caso, a queixa do paciente foi inchaço com rápido crescimento na área cervical, facilitando o diagnóstico.

Um dos principais desafios é diferenciar o OA do osteossarcoma, com um quarto dos casos tendo características radiológicas de malignidade, semelhantes ao osteossarcoma de baixo grau. Há tanta sobreposição de características histológicas e radiológicas que o curso clínico desempenha um papel crítico. 4

Os osteoblastomas são geralmente histologicamente indistinguíveis dos osteomas osteoides, sendo estes raramente maiores que 1,5 cm. As taxas de incidência na coluna vertebral são de 40%, geralmente comprometendo os pedículos e a lâmina. Por outro lado, o OA é associado mais frequentemente com a extensão paravertebral e peridural.

Osteoblastoma agressivo também pode ser semelhante a cistos ósseos aneurismais, osteossarcomas e metástases ósseas, apresentando um padrão expansível com calcificação matricial e destruição óssea cortical. 2

Os osteoblastomas são, geralmente, lesões radiolúcidas, mal definidas com destruição cortical ou bordas bem-definidas e diferentes graus de mineralização. 5 Histopatologicamente, o OA apresenta proeminência nucleolar, trabécula maior e irregular, citoplasma eosinofílico e tendência a ter células mais parecidas com osteoclastos. Pleomorfismo celular leve e mitoses são observados mais frequentemente. 2

Geralmente, o tratamento desta forma agressiva de doença é baseado na ressecção ampla e completa do tumor primário. A complicação adicional ocorre quando o local primário está próximo de estruturas nobres, como neste caso, com o envolvimento da coluna cervical. Uma ressecção radical e completa ainda é considerada o padrão de cuidado, 6 mas há relatos de casos da eficácia potencial da radioterapia, sendo 25 anos o maior tempo de remissão publicado. Um tratamento bem sucedido com curetagem e radioterapia é descrito em um caso em que a ressecção não foi considerada possível. Janin et al. 7 afirmam que a radioterapia deve ser considerada apenas para pacientes cujas lesões são irressecáveis. Há alguns relatos de casos associando a radioterapia ao OA com o desenvolvimento de sarcoma, levando à morte, em último caso. 8 9 Essa complicação preocupante é agravada quando a radioterapia é utilizada especialmente em lesões benignas.

O presente relatório demonstra uma demanda clínica por ensaios randomizados em doenças raras. Existem vários projetos e esforços que podem ser feitos para superar o problema natural de testar novos tratamentos no contexto de doenças raras. 2 Este relatório acrescenta aos dados clínicos que a radioterapia é um tratamento potencial para o osteoblastoma agressivo. Além disso, há relatos de casos com recidiva do osteoblastoma após radioterapia semelhante a este caso. 10 O tratamento até agora bem-sucedido destaca a importância de uma abordagem multidisciplinar para o tratamento desses pacientes, especialmente em caso de doenças raras.
